# Prognostic Value of Fibrosis-4 Index, Hepatic Biomarkers, and Plasma Acute-Phase Reactants in Critical Patients Undergoing Percutaneous Endoscopic Gastrostomy: A Retrospective Cohort Study

**DOI:** 10.3390/jcm15135084

**Published:** 2026-06-30

**Authors:** Kubilay İşsever, Ali Muhtaroğlu, Ersin Kuloğlu, Sefer Aslan, Berkan Acar, Mehmet Yıldız, Gökhan Aydın, Kamil Konur, Ahmet Cumhur Dülger

**Affiliations:** 1 Department of Internal Medicine, Division of General Internal Medicine, Faculty of Medicine, Giresun University, 28200 Giresun, Türkiye; kubilayissever@gmail.com (K.İ.); ersinkuloglu.28@hotmail.com (E.K.); drseferaslan02@hotmail.com (S.A.); 2 General Surgery Clinic, Private Adatıp Hospital, 54050 Sakarya, Türkiye; alimuhtarogluu@gmail.com; 3 Department of General Surgery, Faculty of Medicine, Giresun University, 28200 Giresun, Türkiye; berkanacar01@gmail.com; 4 Giresun Provincial Health Directorate, Bulancak Sehit Er Enver Erdogan Family Health Center, 28300 Giresun, Türkiye; drmehmetyildiz54@gmail.com; 5 Division of Gastroenterology, Department of Internal Medicine, Faculty of Medicine, Giresun University, 28200 Giresun, Türkiye; g.aydin@giresun.edu.tr (G.A.); acdulger@gmail.com (A.C.D.); 6 Department of Internal Medicine, Division of General Internal Medicine, Faculty of Medicine, Recep Tayyip Erdogan University, 53200 Rize, Türkiye

**Keywords:** prognostic indicators, percutaneous endoscopic gastrostomy, intensive care unit, hepatic biomarkers, FIB-4 score

## Abstract

**Background/Objectives**: Identifying reliable predictors of all-cause mortality in intensive care unit (ICU) patients undergoing percutaneous endoscopic gastrostomy (PEG) remains clinically important to decide the route of nutrition. This study aimed to assess the prognostic significance of hepatic biomarkers, particularly the Fibrosis-4 (FIB-4) score. **Methods**: We conducted a retrospective cohort study in the ICUs of a tertiary care university hospital. Adult patients who underwent PEG between 1 January 2022 and 31 December 2024 were included. Pre-procedural demographic, clinical, and laboratory data were retrieved from electronic medical records. The primary outcome was all-cause mortality within three years of PEG placement. Survival analyses were performed using Kaplan–Meier curves and Cox proportional hazards regression to identify independent predictors. **Results**: Older age, elevated FIB-4, total and direct bilirubin, gamma glutamyl transferase (GGT), lactate dehydrogenase (LDH), ferritin, c-reactive protein (CRP), procalcitonin, INR, and prothrombin (PT) levels, and reduced calcium, platelet count, and albumin were significantly associated with all-cause mortality (*p* < 0.05). ROC analysis identified FIB-4, GGT, LDH, and albumin as significant predictors of mortality. Kaplan–Meier analysis confirmed that patients with higher FIB-4, GGT, and LDH, and lower albumin levels, had significantly shorter survival. Cox proportional hazards regression analysis revealed that while the FIB-4 score was a significant predictor of mortality in the univariate model, it lost its independent prognostic value when adjusted for confounders. Instead, lower albumin, elevated CRP, and increased GGT emerged as the independent risk factors for mortality. **Conclusions**: Pre-procedural albumin, CRP, and GGT levels are strong independent prognostic indicators of all-cause mortality in ICU patients undergoing PEG. While FIB-4 serves as a practical initial screening tool, adverse outcomes might be primarily driven by systemic inflammation and nutritional depletion rather than isolated hepatic fibrosis.

## 1. Introduction

Percutaneous endoscopic gastrostomy (PEG) is a procedure commonly performed in critically ill patients who cannot maintain adequate oral intake, providing long-term enteral nutrition. Although PEG is generally considered a safe procedure, the outcomes for patients in the intensive care unit (ICU) can vary significantly. These variations depend on several factors, including underlying health conditions, nutritional status, and physiological complications [1]. For example, ICU patients with advanced liver dysfunction or coexisting organ failure may be at greater risk for PEG-related complications and poorer outcomes, particularly when hypoalbuminemia and inflammation coexist [2]. A recent study by Erdemir et al. demonstrated that nearly one-quarter of ICU patients undergoing PEG experienced minor procedure-related complications, and over half of the cohort died within a maximum follow-up of 52 weeks. These findings further highlight the ongoing controversy in the literature regarding the optimal timing and patient selection for PEG in the intensive care setting [3]. Therefore, it is crucial to identify reliable prognostic markers before PEG placement to guide clinical decision-making and enhance outcomes for this high-risk population.

Recent investigations have increasingly focused on hepatic and metabolic biomarkers as predictors of poor outcomes in ICU settings. Traditional liver function tests, such as ALT/AST, bilirubin, and GGT, have been associated with higher mortality rates in critically ill patients [4,5]. The Fibrosis-4 (FIB-4) index, initially developed to estimate hepatic fibrosis in patients with viral hepatitis, has been shown to predict in-hospital and 1-year mortality in ICU patients, particularly those with cardiovascular comorbidities [6,7]. Additionally, routinely measured laboratory parameters, including LDH and albumin, may indicate systemic inflammation, cellular injury, and nutritional depletion—factors that are particularly relevant in the ICU environment [8].

To date, no published study has thoroughly examined the prognostic value of pre-procedural FIB-4, GGT, LDH, and albumin levels specifically in ICU patients undergoing PEG. Therefore, this retrospective cohort study aimed to assess the relationship between these biomarkers and one-year all-cause mortality following PEG placement in ICU patients. The objective is to identify practical, low-cost predictors that can aid in individualized risk stratification and management.

## 2. Materials and Methods

### 2.1. Study Design and Data Collection

We report a single-center retrospective cohort study of PEG consultations sent from the intensive care units of our hospital to the endoscopy unit between 1 January 2022 and 31 December 2024, with a minimum follow-up period of one month. Data from all PEG patients were subsequently collected. PEG placement indications were applied to all ICU patients according to the following recommendation of the ESPEN 2023 guidelines: “PEG tube placement is indicated in ICU patients who are expected to require enteral feeding for more than 4–6 weeks and have a functioning gastrointestinal tract but are unable to meet their nutritional requirements orally.” [9]. Patients who were discharged or referred without follow-up after PEG application, patients in whom enteral nutrition failed despite PEG placement, nephrotic patients with albuminuria, patients with a history of parenchymal liver disease other than steatotic liver disease (SLD), patients using hepatotoxic agents or receiving medical treatment that may cause fluctuations in liver enzyme levels, patients with active or chronic hepatitis, and patients under 18 years of age were excluded from the study. Informed consent was not obtained due to the retrospective nature of the study. This study was conducted in accordance with the ethical principles of the Declaration of Helsinki revised in 2013.

The demographic characteristics and laboratory and clinical data of these patients were collected during consultations for the PEG procedure and analysed retrospectively. Patients recommended for the PEG procedure are usually those who have been in the intensive care unit for a prolonged period due to conditions such as acute myocardial infarction, cerebrovascular accident, pneumonia, or malignancy. This includes individuals who are intubated, have a tracheostomy, are using a nasojejunal tube for enteral feeding, or need enteral feeding for over 30 days. The primary endpoint of this study was all-cause mortality following PEG placement. The survival status, follow-up duration (in months), and exact dates of death for all patients, including those who died after hospital discharge, were reliably obtained using the National Electronic Health Records and Death Reporting System. Patients were followed from the date of PEG placement until death or the end of the study’s data collection period. Based on these records, patients who died at any time during the follow-up period were classified as ‘non-survivors’, whereas those who remained alive at the end of the follow-up were classified as ‘survivors’.

The SLD data of the patients were presented according to the abdominal ultrasonography (USG) findings, routinely performed by the same radiologist before the PEG procedures, since transient elastography was not available at our hospital. Regarding the degree of SLD, patients with grades 1 and 2 were classified as “SLD present,” and those without SLD were classified as “SLD absent.” All laboratory tests used in the statistical analysis, as well as FIB-4 levels, were withdrawn and calculated at the time of the patient’s ICU admission. As recommended in the guidelines, a FIB-4 score of <1.3 indicates low fibrosis risk, a FIB-4 score between 1.3 and 2.67 indicates moderate fibrosis risk, and a FIB-4 score > 2.67 indicates high fibrosis risk [10].

### 2.2. Application of the PEG Procedure

The technique of PEG tube placement was performed in line with the British Society of Gastroenterology (BSG) practice guidelines [11]. The same gastroenterologist performed PEG tube insertion using the pull technique under sterile conditions. Thirty minutes before the interventional procedure, 2 g of ceftriaxone was administered intravenously as prophylaxis. Depending on the patient’s condition, midazolam and propofol were administered in weight-adjusted doses when sedation was required. After a skin shave, a 1-cm skin incision was made before PEG insertion in all patients with positive transillumination. The PEG tube insertion was performed using the PEG 24^®^ Pull Method™ (Cook Medical, Bloomington, IN, USA). After inserting the PEG tube, it was secured with an exterior retention plate without sutures. The dressing was changed three times a day for the first 7 days after the procedure, and water was given through the PEG tube 24 h after placement. Initially, 100 mL of oral nutritional supplement (ONS) was administered if there were no complications following the PEG procedure. If this was tolerated, an additional 50 mL of ONS was added to the previous volume, as described by Jung et al. [12].

### 2.3. Statistical Analysis

The data were analysed using the IBM SPSS Statistics for Windows, version 26.0 (IBM Corp., Armonk, NY, USA). The descriptive characteristics of patients who underwent PEG were presented as a number and percentage. Non-normally distributed data were presented as median, minimum, and maximum, while normally distributed data were presented as mean, standard deviation, median, minimum, and maximum. The suitability of the data for normal distribution was determined by examining the skewness and kurtosis values. In accordance with standard practice, the reference value for normal distribution is between ±1.96. All other variables did not meet the assumptions of normality except for age, cholesterol, HDL, LDL, ferritin, total protein, albumin, D-dimer, fibrinogen, leucocyte, and sodium. A Chi-Square Test was employed to evaluate the correlation between mortality and three variables: gender, the presence of SLD, and FIB-4 groups. Various numerical parameters concerning mortality were compared using an Independent Sample T-test for data that followed a normal distribution.

Furthermore, the Mann–Whitney U test was employed to compare the various numerical parameters concerning mortality in non-normally distributed data. The relationships between mortality and all other variables were analysed using Spearman’s correlation coefficient, as appropriate for non-normally distributed data and ordinal variables. The correlation coefficient was evaluated as indicating a low-level relationship (0.00–0.30), a medium-level relationship (0.30–0.70), or a high-level relationship (0.70–1.00). A Receiving Operator Characteristic (ROC) analysis was conducted to assess mortality risk in patients based on PEG duration, FIB-4, GGT, LDH, and albumin levels [13]. The ROC analysis demonstrates that the area under the ROC curve is a statistically significant predictor of both mortality risk and survival probability in patients. The ROC analysis revealed that when there is no ability to discriminate between the probability of patients who died or survived, the expected value of the area under the ROC curve is 0.50. In a perfect test, these values are 1.00. The values under the curve are interpreted as follows: 0.90–1.00 = excellent, 0.80–0.90 = good, 0.70–0.80 = moderate, 0.60–0.70 = poor, and 0.50–0.60 = failure. Throughout the study, significance levels of 0.05 and 0.01 were maintained. The impact of these variables on mortality risk, based on the determined cut-off values, was investigated using Kaplan–Meier survival analysis. Furthermore, a Cox proportional hazards regression analysis was performed to identify independent risk factors associated with mortality in patients undergoing PEG. The results were presented using hazard ratios (HR), 95% confidence intervals (CI), and *p*-values. The discriminative performance of the model was evaluated using Harrell’s C-index.

## 3. Results

### 3.1. Demographic and Laboratory Results

The study was conducted by analysing the data of 149 patients, comprising 88 in the survivor group and 61 in the non-survivor group. The mean age of the survivor group was significantly lower than that of the non-survivor group (72.94 ± 18.52 vs. 80.11 ± 15.63). The distributions of gender and SLD presence were not significantly different between the groups. However, there was a significant difference in the distribution of FIB-4 levels according to mortality status (*p* < 0.05). These data show that the proportion of patients with low fibrosis risk was significantly higher in the survivor group (52.3% vs. 24.6%). The non-survivor group had a significantly higher rate of high fibrosis risk compared to the survivor group (44.3% vs. 10.2%). The distribution of patients who underwent PEG according to demographic and disease characteristics and the presence of mortality are shown in Table 1.

There was no significant difference regarding indirect bilirubin, alkaline phosphatase (ALP), amylase, ALT, total protein, glucose, lactate, cholesterol, triglyceride, HDL cholesterol, LDL cholesterol, D-dimer, fibrinogen, leucocyte, sodium, and potassium levels between the groups. However, AST, total bilirubin, direct bilirubin, GGT, LDH, ferritin, CRP, procalcitonin, INR, and PT levels were significantly higher (*p* = 0.03, 0.002, 0.001, 0.02, 0.000, 0.001, 0.000, 0.01, 0.001, 0.01, respectively) in non-survivors. In contrast, albumin, platelet, and calcium levels were lower in the same group (*p* = 0.000, 0.01, 0.02, respectively). Laboratory parameters of the groups are compared in Table 2.

### 3.2. Correlation Analysis

Age, FIB-4 score, AST, total bilirubin, direct bilirubin, GGT, LDH, CRP, INR, and PT levels showed low and moderate positive correlations with mortality. Albumin, platelet, and calcium levels showed low and moderate negative correlations with mortality. No correlation was observed between other parameters and mortality. The results of the correlation analysis between mortality and the group parameters are presented in Table 3.

### 3.3. ROC Analysis

**Figure 1:**
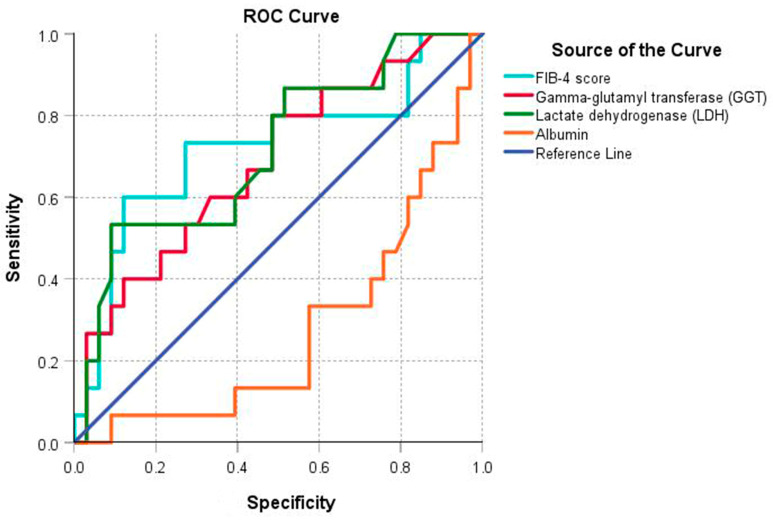
Receiver Operating Characteristic (ROC) curve analysis of FIB-4, GGT, LDH, and albumin levels for predicting mortality.

The ROC analysis revealed that FIB-4, GGT, and LDH levels were significant predictors of death, and albumin level was a significant predictor of survival probability (*p* = 0.01, 0.04, 0.02, and 0.000, respectively). The FIB-4 score, GGT, and LDH levels were moderately effective in predicting mortality risk, while albumin levels were moderately effective in predicting survival probability. The FIB-4 cut-off value was 1.50, with a sensitivity of 73% and a specificity of 58%. The cut-off values for both GGT and LDH were 28.50 and 194.50, respectively. Both variables showed sensitivity and specificity values of 80% and 52%, respectively. The cut-off value for albumin was 28.20, with a sensitivity of 63% and a specificity of 73%. When we compared the predictive ability of the FIB-4 score, GGT, and LDH levels for mortality, FIB-4 was superior to GGT and LDH levels (AUC: 0.72, 0.69, and 0.71, respectively). The results of the ROC analysis are shown in Table 4 and Figure 1.

### 3.4. Kaplan–Meier Survival Analysis

**Figure 2:**
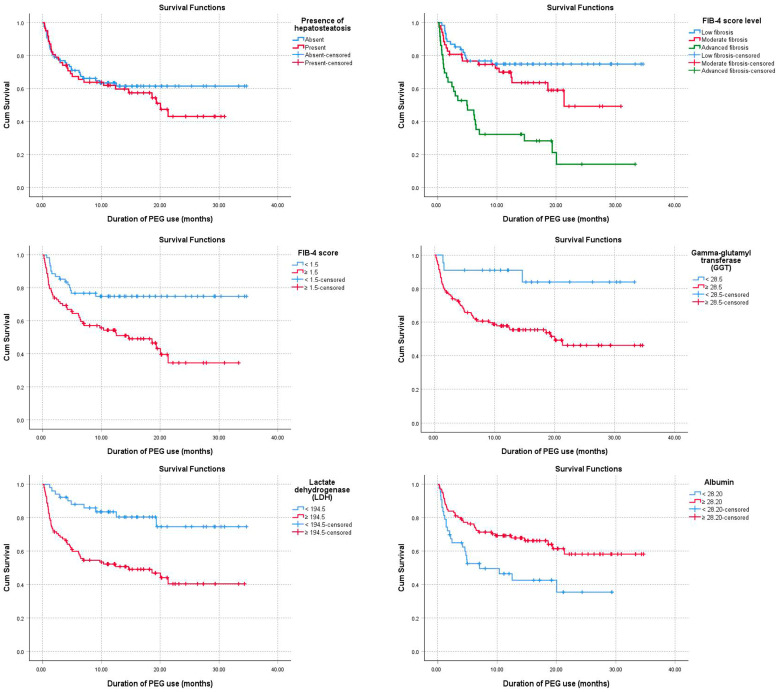
Kaplan-Meier Analysis of Effects of Various Variables on Mortality Risk.

Kaplan–Meier survival analysis was performed to evaluate differences in survival time by key prognostic markers. Patients with FIB-4 scores ≥ 1.5, GGT ≥ 28.5 U/L, LDH ≥ 194.5 U/L, and albumin < 28.2 g/dL had significantly shorter survival than those without these features (log-rank *p* < 0.05 for all). Additionally, patients with higher fibrosis risk have a higher likelihood of mortality, according to this analysis. Table 5 and Figure 2 show the effects of different variables on mortality risk using Kaplan–Meier survival analysis.

### 3.5. Cox Proportional Hazards Regression Analysis

To identify independent risk factors associated with mortality in patients undergoing PEG, a Cox proportional hazards regression analysis was performed (Table 6). Variables were selected based on clinical relevance and univariate analysis results. In the univariate Cox regression analysis, age (HR = 1.02; 95% CI: 1.00–1.04; *p* = 0.025), FIB-4 score (HR = 1.10; 95% CI: 1.05–1.15; *p* < 0.001), albumin (HR = 0.91; 95% CI: 0.87–0.96; *p* < 0.001), and CRP (HR = 1.00; 95% CI: 1.00–1.01; *p* = 0.018) were significantly associated with increased mortality. However, in the multivariate Cox regression model—which evaluated age, FIB-4 score, albumin, CRP, and GGT together—albumin (HR = 0.91; 95% CI: 0.82–1.00; *p* = 0.041), CRP (HR = 1.01; 95% CI: 1.00–1.01; *p* = 0.023), and GGT (HR = 1.01; 95% CI: 1.00–1.01; *p* = 0.008) remained independent predictors of mortality. Notably, when adjusted for these clinical confounders, age and the FIB-4 score lost their statistical significance (*p* = 0.746 and *p* = 0.164, respectively). The Harrell C-index for the model was 0.651, indicating moderate discriminative power for predicting mortality risk.

## 4. Discussion

In this retrospective study, we identified several pre-procedural hepatic and biochemical parameters linked to all-cause mortality among ICU patients undergoing percutaneous endoscopic gastrostomy (PEG). Predicting mortality in this highly vulnerable population, particularly among elderly ICU patients, remains a critical clinical challenge [14,15]. Our findings indicate that FIB-4, GGT, LDH, and albumin—biomarkers that are readily available and cost-effective—can provide valuable prognostic information for this high-risk population. These biomarkers may help clinicians effectively stratify risk and make informed clinical decisions, particularly regarding the suitability of PEG placement for patients.

An important initial finding of our study was the strong association between elevated FIB-4 scores and mortality rates in the univariate analysis. However, in the multivariate Cox regression model, the FIB-4 score lost its independent predictive value when adjusted for age, albumin, CRP, and GGT. This is a critical pathophysiological finding for the intensive care setting. The loss of FIB-4’s independent predictive value can be largely explained by the inherent mathematical nature of its calculation, which fundamentally incorporates age and platelet count. In our cohort, non-survivors were significantly older and had notably lower platelet counts compared to survivors. Since advanced age and thrombocytopenia—often indicators of severe sepsis, systemic inflammation, or multi-organ failure in the ICU—are directly and strongly associated with mortality, their heavy mathematical weight in the formula artificially inflates the FIB-4 score in dying patients. Consequently, this disrupts the objectivity of FIB-4 as a specific surrogate for chronic hepatic fibrosis. As highlighted in the recent literature, while FIB-4 is an established marker of chronic hepatic fibrosis in stable patients, its fluctuations in critically ill patients likely reflect acute systemic inflammation, multi-organ dysfunction, and hemodynamic instability rather than true chronic liver architecture [16,17,18]. Our results mathematically support this phenomenon: the mortality risk in these patients is fundamentally driven by severe systemic inflammation (reflected by CRP), advanced age, and profound physiological and nutritional depletion (reflected by hypoalbuminemia), rather than isolated hepatic fibrosis [19,20].

Beyond the FIB-4 score which has been previously associated with adverse outcomes in various critical care populations our multivariate analysis identified elevated CRP and GGT, alongside hypoalbuminemia, as the true independent prognostic markers for mortality in ICU patients undergoing PEG [16,17,18]. Although parameters like LDH and ferritin showed initial associations with adverse outcomes, their predictive power diminished after adjusting for the patients’ inflammatory and nutritional baselines. The independent prognostic value of GGT in this setting is particularly noteworthy. Rather than just indicating biliary issues, an elevated GGT in critically ill patients often reflects profound oxidative stress and ongoing cellular damage. Similarly, CRP serves as a direct indicator of the acute-phase response and systemic inflammatory burden. Conversely, higher serum albumin levels provided a strong protective effect. Since albumin is a reliable reflection of physiological and nutritional reserves, its depletion in the non-surviving group strongly supports the idea that mortality after PEG placement is driven more by baseline frailty and active inflammation than by isolated structural liver dysfunction [19,20].

Translating these prognostic insights into bedside practice directly addresses the clinical dilemma of deciding the appropriate nutritional route for critically ill patients. Although PEG is a beneficial procedure to maintain enteral nutrition, especially for the patients in critical conditions, it also has complication risks, requires a sensitive follow-up period and caregivers who has high sociocultural and medical awareness levels [21,22,23]. When a clinician encounters an ICU patient referred for PEG who exhibits severe hypoalbuminemia coupled with elevated CRP and GGT levels (often accompanied by an artificially inflated FIB-4 score), this biochemical profile signals an overwhelming acute inflammatory state and a critically depleted physiological reserve. In such high-risk scenarios, proceeding immediately with an invasive PEG placement might expose the patient to unnecessary procedural risks with little potential for long-term benefit. Instead, the actionable alternative would be to temporarily postpone the PG procedure. Clinicians should consider maintaining enteral feeding via a less invasive nasogastric or nasojejunal tube, or initiating short-term total parenteral nutrition (TPN) if enteral routes are contraindicated. Once the acute inflammatory phase subsides and the patient’s nutritional baseline improves, the suitability for PEG can be safely re-evaluated. This biomarker-guided approach helps prevent futile invasive procedures in patients whose immediate mortality risk is predominantly driven by active systemic illness rather than anatomical factors.

### Limitations

Several limitations should be acknowledged. First, the retrospective single-center design may introduce selection bias and limit the generalizability of the findings. While the sample size is adequate for this cohort analysis, the results require validation in larger prospective studies. Second, we relied on single time-point (pre-procedural) laboratory values and did not evaluate the longitudinal, day-to-day dynamic fluctuations of these biomarkers during the entire ICU stay. Third, although we adjusted for major confounders, we could not account for all dynamic ICU variables, such as specific daily ICU severity scores (e.g., APACHE II or SOFA) and detailed changes in daily nutritional interventions. Furthermore, we could not assess specific PEG-related complications during the long-term follow-up, all due to the retrospective design of the study. Moreover, our ROC analyses demonstrated moderate discriminatory performance (AUC values ranging from 0.69 to 0.74), highlighting that these biomarkers provide modest prognostic information and should be utilized as adjunctive tools within a comprehensive clinical evaluation rather than as standalone predictive instruments. Finally, due to limited availability and the critical condition of the patients, we assessed hepatic steatosis using ultrasonography instead of transient elastography or histological confirmation.

## 5. Conclusions

In conclusion, our findings indicate that lower serum albumin, elevated CRP, and increased GGT levels serve as strong, independent prognostic markers for all-cause mortality among ICU patients undergoing PEG. Although the FIB-4 index initially appears as a significant predictor, its value is heavily confounded by advanced age and acute systemic inflammation, limiting its utility as an independent marker in the intensive care setting. Consequently, clinical decisions regarding long-term enteral access should not be based merely on isolated liver fibrosis scores. A more comprehensive evaluation that incorporates the patient’s active inflammatory burden and nutritional reserve is essential. By integrating these routine biochemical parameters into daily assessments, clinicians can better stratify high-risk patients, tailor nutritional interventions, and ultimately avoid potentially futile procedures in highly vulnerable individuals.

## Figures and Tables

**Table 1:** The comparison of demographic data and comorbidity characteristics of the groups.

Patients and Disease Characteristics		Survivors (*n*: 88)		Non-Survivors (*n*: 61)		*p*
		*n*	%	*n*	%	
Gender	Female	47	53.4	31	50.8	0.89
	Male	41	46.6	30	49.2	
SLD	No	55	62.5	32	52.5	0.29
	Yes	33	37.5	29	47.5	
FIB-4 level	Low fibrosis risk	46	52.3	15	24.6	0.000 **
	Moderate fibrosis risk	33	37.5	19	31.1	
	High fibrosis risk	9	10.2	27	44.3	
		Med. ± S.D. (Min.–Max.)		Med. ± S.D. (Min.–Max.)		*p*
Age ^t^		72.94 ± 18.52 (19–98)		80.11 ± 15.63 (25–98)		0.01 *
Number of co-morbidities ^t^		2.98 ± 1.39 (1–6)		3.00 ± 1.21 (1–5)		0.92
FIB-4 score ^z^		2.07 ± 2.44 (0.1–8.06)		4.07 ± 4.59 (0.13–25.92)		0.000 **

Footnotes: Bold values indicate statistically significant differences. Abbreviations: * *p* < 0.05; ** *p* < 0.01, Categorical data: Chi-Square Test, ^t^: Independent Sample *t*-Test, ^z^: Mann–Whitney U Test, Med.: Median, S.D.: Standard deviation, Min.: Minimum, Max.: Maximum FIB-4: Fibrosis-4.

**Table 2:** The comparison of laboratory parameters between the groups.

Parameters	Survivors (*n*: 88)	Non-Survivors (*n*: 61)	*p*
	Med. (Min.–Max.)	Med. (Min.–Max.)	
ALT(U/L) ^z^	18 (3–344)	16 (5–1334)	0.711
AST(U/L) ^z^	22 (6–1156)	27 (5–1532)	0.03 *
Total bilirubin (mg/dL) ^z^	0.40 (0.15–5.81)	0.51 (0.17–5.96)	0.002 **
Direct bilirubin(mg/dL) ^z^	0.17 (0.05–5.75)	0.28 (0.09–5.26)	0.001 **
Indirect bilirubin(mg/dL) ^z^	0.14 (0.03–2.05)	0.22 (0.06–0.52)	0.139
ALP (IU/L) ^z^	106.5 (45–453)	110 (62–641)	0.686
GGT (IU/L) ^z^	24 (61–454)	46 (12–306)	0.02 *
LDH (U/L) ^z^	206.5 (120–5628)	285.5 (130–1688)	0.000 **
Amylase (IU/L) ^z^	50 (3–290)	58.5 (12–535)	0.352
Ferritin (ng/mL) ^t^	154.95 (11.9–2000)	1463 (124.5–2000)	0.001 **
Total Protein (gr/dL) ^t^	64.68 (48.16–82.47)	62.4 (46.61–72.55)	0.146
Albumin (gr/dL) ^t^	31.3 (19.5–48)	25.3 (13.8–42.5)	0.000 **
Glucose (mg/dL) ^z^	114 (62–413)	127 (26–361)	0.085
Lactate (mmol/L) ^z^	1.3 (0.2–13.1)	1.5 (0.5–16.6)	0.194
Total cholesterol (mg/dL) ^t^	146 (53–330)	153 (58–285)	0.869
Triglyceride (mg/dL) ^z^	107 (34–490)	109.5 (35–430)	0.795
HDL cholesterol (mg/dL) ^t^	37 (14–79)	39 (15–73)	0.319
LDL cholesterol (mg/dL) ^t^	88 (13–219)	86 (20–192)	0.909
CRP ^z^	49.79 (0.28–447.78)	95.25 (2.21–410.29)	0.000 **
Procalcitonin(ng/mL) ^z^	0.21 (0.03–51.29)	1.05 (0.05–100)	0.01 *
D-dimer(ng/mL) ^t^	2873.5 (1556–4998)	3195.5 (194–9925)	0.281
INR ^z^	1.07 (0.89–7.86)	1.15 (0.92–3.44)	0.001 **
PT ^z^	9.53 (8.05–81)	10.75 (8.2–34)	0.010 **
Fibrinogen (mg/dL) ^t^	524 (155–68)	537 (114–881)	0.405
Leucocyte (10^3^ uL) ^t^	8.91 (0.16–26.62)	9.44 (4.29–30.17)	0.055
Platelet(10^3^/uL) ^z^	288 (27–1176)	213 (49–629)	0.01 **
Calcium(mg/dL) ^z^	9 (6.7–13.9)	8.5 (4–10.7)	0.02 *
Sodium (mEq/L) ^t^	139 (123–151)	139 (122–161)	0.385
Potassium (Eq/L) ^z^	4.2 (2.8–6.2)	4.3 (2.6–10.1)	0.785

Footnotes: Bold values indicate statistically significant differences. Abbreviations: * *p* < 0.05; ** *p* < 0.01; ^t^: Independent Sample *t*-Test, ^z^: Mann–Whitney U Test, Med.: Median, Min.: Minimum, Max.: Maximum ALT: Alanine Aminotransferase, AST: Alkaline aminotransferase, ALP: Alkaline phosphatase, GGT: Gamma-glutamyl transferase, LDH: Lactate dehydrogenase, HDL: High-density lipoprotein, LDL: Low-density lipoprotein, CRP: C-reactive protein, INR: International Normalized Ratio, PT: Prothrombin time.

**Table 3:** The results of the correlation analysis between the presence of mortality and the parameters of the groups.

Variables	Mortality	Variables	Mortality
Gender	0.02	Albumin	−0.40 **
Age	0.22 **	Glucose	0.14
SLD	0.10	Lactate	0.12
FIB-4 level	0.37 **	Total cholesterol	0.02
FIB-4 score	0.35 **	Triglyceride	0.02
Number of comorbidities	0.02	HDL cholesterol	0.08
ALT	−0.03	LDL cholesterol	−0.01
AST	0.18 *	CRP	0.29 **
Total bilirubin	0.25 **	Procalcitonin	0.29 *
Direct bilirubin	0.27 **	D-dimer	0.06
Indirect bilirubin	0.22	INR	0.29 **
ALP	0.06	PT	0.24 **
GGT	0.32 *	Fibrinogen	0.07
LDH	0.39 **	Leucocyte	0.13
Amylase	0.08	Platelet	−0.23 **
		Calcium	−0.19 *

Footnotes: Bold values indicate statistically significant differences. Abbreviations: * *p* < 0.05; ** *p* < 0.01, ALT: Alanine Aminotransferase, AST: Alkaline aminotransferase, ALP: Alkaline phosphatase, GGT: Gamma-glutamyl transferase, LDH: Lactate dehydrogenase, HDL: High-density lipoprotein, LDL: Low-density lipoprotein, CRP: C-reactive protein, INR: International Normalised Ratio, PT: Prothrombin time.

**Table 4:** The results of the ROC analysis to determine the mortality risk of patients.

Variables	AUC *	Std. Error	*p*	Cut-Off Value	Sensitivity (%)	Specificity (%)	Confidence Interval (95)	
							Lowest	Highest
FIB-4 score	**0.72**	0.09	**0.01**	1.50	0.73	0.58	0.55	0.89
GGT	**0.69**	0.08	**0.04**	28.50	0.80	0.52	0.52	0.85
LDH	**0.71**	0.08	**0.02**	194.50	0.80	0.52	0.55	0.87
Albumin	**0.74**	0.06	**0.000**	28.20	0.63	0.73	0.63	0.85

Footnotes: The albumin variable was used to estimate survival probability, and FIB-4, GGT, and LDH were used to assess the risk of death. Bold values indicate statistically significant differences. Abbreviations: * AUC: Area under the curve. Std.: Standard FIB-4: Fibrosis-4, GGT: Gamma-glutamyl transferase, LDH: Lactate dehydrogenase.

**Table 5:** Kaplan–Meier analysis of the effects of various variables on mortality risk.

Variables		Estimated Mean	S.D.	%95 Confidence Interval		*p*
				Low	High	
Presence of Steatotic Liver Disease	No	22.67	1.69	19.37	25.97	0.41
	Yes	17.92	1.73	14.52	21.32	
FIB-4 levels	Low fibrosis risk	26.73	1.78	23.25	30.22	0.000 **
	Moderate fibrosis risk	19.94	1.97	16.07	23.80	
	High fibrosis risk	9.91	2.08	5.84	13.99	
FIB-4 score	Below 1.5	26.73	1.78	23.25	30.22	0.001 **
	Above 1.5	16.48	1.66	13.22	19.74	
GGT	Below 28.5	29.13	2.25	24.72	33.54	**0.01**
	Above 28.5	19.44	1.46	16.58	22.29	
LDH	Below 194.5	28.09	1.86	24.45	31.73	0.001 **
	Above 194.5	17.35	1.63	14.16	20.55	
Albumin	Below 28.2	13.83	2.05	9.80	17.85	**0.01**
	Above 28.2	23.14	1.52	20.16	26.12	

Footnotes: Bold values indicate statistically significant differences. Abbreviations: ** *p* < 0.01, S.D.: Standard deviation, *p*: Log Rank (Mantel–Cox) GGT: Gamma-glutamyl transferase LDH: Lactate dehydrogenase.

**Table 6:** Cox Proportional Hazards Regression Analysis of Factors Predicting Mortality.

Variables	Univariate Cox Regression			Multivariate Cox Regression		
	*p*	HR	%95 CI min–max	*p*	HR	%95 CI min–max
Age	0.025 *	1.02	1.00–1.04	0.746	1.00	0.98–1.03
Number of comorbidities	0.997	1.00	0.83–1.21	-	-	-
FIB-4 score	<0.001 **	1.10	1.05–1.15	0.164	1.07	0.97–1.18
Albumin	<0.001 **	0.91	0.87–0.96	0.041 *	0.91	0.82–1.00
CRP	0.018 *	1.00	1.00–1.01	0.023 *	1.01	1.00–1.01
Lactate dehydrogenase (LDH)	0.242	1.00	1.00–1.00	**-**	-	-
Gamma-glutamyl transferase (GGT)	0.086	1.00	1.00–1.01	0.008 **	1.01	1.00–1.01
INR	0.864	1.03	0.76–1.40	-	-	-

Footnotes: Bold values indicate statistically significant differences. Abbreviations: HR: Hazard Ratio; CI: Confidence Interval. The multivariate model included age, FIB-4 score, albumin, CRP, and GGT. * *p* < 0.05; ** *p* < 0.01. C-index: 0.651. CRP: C-reactive protein. INR: International Normalized Ratio.

## Data Availability

The data presented in this study are available upon reasonable request from the corresponding author. The data are not publicly available due to privacy and ethical re-strictions.

## Abbreviations

The following abbreviations are used in this manuscript:

ALT	Alanine Aminotransferase
AST	Aspartate Aminotransferase
CRP	C-Reactive Protein
FIB-4	Fibrosis-4
GGT	Gamma Glutamyl Transferase
HDL	High-Density Lipoprotein
ICU	Intensive Care Unit
INR	International Normalised Ratio
LDH	Lactate Dehydrogenase
LDL	Low-Density Lipoprotein
MASLD	Metabolic Dysfunction-Associated Steatotic Liver Disease
ONS	Oral Nutritional Supplement
PEG	Percutaneous Endoscopic Gastrostomy
ROC	Receiver Operating Characteristic
SD	Standard Deviation
SLD	Steatotic Liver Disease
SPSS	Statistical Package for the Social Sciences
USG	Ultrasonography
